# Correction: Wang et al. Generalized SCF Formula of Out-Of-Plane Gusset Welded Joints and Assessment of Fatigue Life Extension by Additional Weld. *Materials* 2021, *14*, 1249

**DOI:** 10.3390/ma14092331

**Published:** 2021-04-30

**Authors:** Yixun Wang, Yuxiao Luo, Yuki Kotani, Seiichiro Tsutsumi

**Affiliations:** 1 Joining and Welding Research Institute, Osaka University, Osaka 567-0047, Japan; yuki.kotani@kawada.co.jp; 2 Department of Structural Engineering, Tongji University, Shanghai 200092, China; 1410210@tongji.edu.cn

The authors wish to revise in the text of Appendix A, pages 19–21 [1], due to the presence of incorrect information.

The correct text is shown below:

## Appendix A

Formulae for calculating SCFs for gusset welded joints with a single attachment and double attachments under tensile and bending stress.

(1) Gusset welded joints with a single attachment under tensile stress:

(A1) $$K_{t}=1+2.754886\times 10^{-5}\cdot f(r_{1}/t)\cdot f(\theta_{1})\cdot f\left(\left(r_{1}/t)\cdot (\theta_{1}\right)\right)\cdot f(T/t)\cdot f(L_{1}/t)\cdot f(L_{2}/t)\cdot f(W/t)$$

where:

$$\begin{array}{l} f(r_{1}/t)=-1.384329\cdot {\left(r_{1}/t\right)}^{0.3978921}+8.598751\cdot {\left(r_{1}/t\right)}^{3}-7.659145\cdot {\left(r_{1}/t\right)}^{2}+3.384387\cdot (r_{1}/t)+5.501032\times 10^{-1}\\ f(\theta_{1})=-2.731085\times 10^{-1}\cdot {\left(\theta_{1}\right)}^{3}-1.305053\times 10^{-1}\cdot {\left(\theta_{1}\right)}^{2}+3.523407\cdot (\theta_{1})+2.808286\\ \begin{array}{ll} f\left(\left(r_{1}/t)\cdot (\theta_{1}\right)\right)= & 3.608792\cdot {\left(\left(r_{1}/t)\cdot (\theta_{1}\right)\right)}^{1.000578}+3.115798\times 10^{-3}\cdot {\left(\left(r_{1}/t)\cdot (\theta_{1}\right)\right)}^{3}-5.948326\times 10^{-3}\cdot {\left(\left(r_{1}/t)\cdot (\theta_{1}\right)\right)}^{2}\\ & -\;3.605840\cdot ((r_{1}/t)\cdot (\theta_{1}))+4.749695\times 10^{-4} \end{array}\\ f(T/t)=-6.706140\times 10^{-2}\cdot {\left(T/t\right)}^{3}+2.808218\times 10^{-1}\cdot {\left(T/t\right)}^{2}-1.092288\cdot (T/t)+8.585715\\ f(L_{1}/t)=-2.300747\times 10^{-1}\cdot {\left(L_{1}/t\right)}^{4}+6.891865\times 10^{-1}\cdot {\left(L_{1}/t\right)}^{3}+1.584404\times 10^{-1}\cdot {\left(L_{1}/t\right)}^{2}-2.585717\cdot (L_{1}/t)+9.521052\\ \begin{array}{ll} f(L_{2}/t)= & 1.879764\cdot {\left(L_{2}/t\right)}^{0.8153879}+1.392864\cdot {\left(L_{2}/t\right)}^{4}-1.792958\cdot {\left(L_{2}/t\right)}^{3}-9.829634\times 10^{-1}\cdot {\left(L_{2}/t\right)}^{2}+7.194295\times 10^{-1}\cdot (L_{2}/t)\\ & +1.447930 \end{array}\\ f(W/t)=-1.278819\times 10^{-1}\cdot {\left(W/t\right)}^{4}+3.057878\times 10^{1}\cdot {\left(W/t\right)}^{3}-2.505313\times 10^{3}\cdot {\left(W/t\right)}^{2}+7.782724\times 10^{4}\cdot (W/t)+9.862398\times 10^{5} \end{array}$$

(2) Gusset welded joints with a single attachment under bending stress:

(A2) $$K_{t}=1+1.089691\times 10^{-4}\cdot f(r_{1}/t)\cdot f(\theta_{1})\cdot f\left(\left(r_{1}/t)\cdot (\theta_{1}\right)\right)\cdot f(T/t)\cdot f(L_{1}/t)\cdot f(L_{2}/t)\cdot f(H/t)\cdot f(W/t)\cdot f\left(\left(T/t)\cdot (L_{1}/t\right)\right)$$

where:

$$\begin{array}{l} f(r_{1}/t)=2.223480\times 10^{-1}\cdot {\left(r_{1}/t\right)}^{-0.9588104}-9.036846\cdot {\left(r_{1}/t\right)}^{3}+9.251138\cdot {\left(r_{1}/t\right)}^{2}-3.922908\cdot (r_{1}/t)+1.291691\\ f(\theta_{1})=-1.245222\times 10^{-1}\cdot {\left(\theta_{1}\right)}^{3}+6.159986\times 10^{-1}\cdot {\left(\theta_{1}\right)}^{2}-1.371642\cdot (\theta_{1})+2.713439\\ \begin{array}{ll} f\left(\left(r_{1}/t)\cdot (\theta_{1}\right)\right)= & 3.672617\cdot {\left(\left(r_{1}/t)\cdot (\theta_{1}\right)\right)}^{0.8179879}-1.709043\cdot {\left(\left(r_{1}/t)\cdot (\theta_{1}\right)\right)}^{3}+2.459607\cdot {\left(\left(r_{1}/t)\cdot (\theta_{1}\right)\right)}^{2}-4.562573\cdot ((r_{1}/t)\cdot (\theta_{1}))\\ & -3.311594\times 10^{-3} \end{array}\\ f(T/t)=1.535657\times 10^{-1}\cdot {\left(T/t\right)}^{3}-2.233236\times 10^{-1}\cdot {\left(T/t\right)}^{2}-3.221371\cdot (T/t)+1.585198\times 10^{1}\\ f(L_{1}/t)=9.147005\times 10^{-2}\cdot {\left(L_{1}/t\right)}^{4}-5.379052\times 10^{-1}\cdot {\left(L_{1}/t\right)}^{3}+1.217043\cdot {\left(L_{1}/t\right)}^{2}-1.372365\cdot (L_{1}/t)+9.554910\times 10^{-1}\\ \begin{array}{ll} f(L_{2}/t)= & 2.323289\cdot {\left(L_{2}/t\right)}^{0.07198295}+1.128889\times 10^{-1}\cdot {\left(L_{2}/t\right)}^{4}-4.608254\times 10^{-1}\cdot {\left(L_{2}/t\right)}^{3}+7.908669\times 10^{-1}\cdot {\left(L_{2}/t\right)}^{2}\\ & -8.168302\times 10^{-1}\cdot (L_{2}/t)-1.947074 \end{array}\\ f(H/t)=-8.109550\times 10^{1}\cdot {\left(H/t\right)}^{4}+4.866448\times 10^{1}\cdot {\left(H/t\right)}^{3}-9.667820\cdot {\left(H/t\right)}^{2}+9.059584\times 10^{-1}\cdot (H/t)+6.694241\times 10^{-1}\\ f(W/t)=-1.353206\times 10^{-1}\cdot {\left(W/t\right)}^{4}+3.728223\times 10^{1}\cdot {\left(W/t\right)}^{3}-3.820716\times 10^{3}\cdot {\left(W/t\right)}^{2}+1.792792\times 10^{5}\cdot (W/t)+1.212907\times 10^{6}\\ \begin{array}{ll} f\left(\left(T/t)\cdot (L_{1}/t\right)\right)= & 5.284976\cdot {\left(\left(T/t)\cdot (L_{1}/t\right)\right)}^{0.7541015}-6.653339\times 10^{-2}\cdot {\left(\left(T/t)\cdot (L_{1}/t\right)\right)}^{3}+5.796792\times 10^{-1}\cdot {\left(\left(T/t)\cdot (L_{1}/t\right)\right)}^{2}\\ & -\;4.395484\cdot \left(\left(T/t)\cdot (L_{1}/t\right)\right)+1.429009 \end{array} \end{array}$$

(3) Gusset welded joints with double attachments under tensile stress:

(A3) $$K_{t}=1-3.220317\times 10^{-8}\cdot f(r_{1}/t)\cdot f(\theta_{1})\cdot f\left(\left(r_{1}/t)\cdot (\theta_{1}\right)\right)\cdot f(T/t)\cdot f(L_{1}/t)\cdot f(L_{2}/t)\cdot f(H/t)\cdot f(W/t)\cdot f\left(\left(T/t)\cdot (L_{1}/t\right)\right)$$

where:

$$\begin{array}{l} f(r_{1}/t)=1.682930\cdot {\left(r_{1}/t\right)}^{-0.1840345}-1.743797\times 10^{1}\cdot {\left(r_{1}/t\right)}^{3}+4.538856\cdot {\left(r_{1}/t\right)}^{2}+2.538109\cdot (r_{1}/t)+2.245881\times 10^{-1}\\ f(\theta_{1})=-1.131578\times 10^{-1}\cdot {\left(\theta_{1}\right)}^{3}-7.136935\times 10^{-1}\cdot {\left(\theta_{1}\right)}^{2}+3.799505\cdot (\theta_{1})+1.307790\\ \begin{array}{ll} f\left(\left(r_{1}/t)\cdot (\theta_{1}\right)\right)= & 3.711756\cdot {\left(\left(r_{1}/t)\cdot (\theta_{1}\right)\right)}^{1.0005805}+3.488500\times 10^{-3}\cdot {\left(\left(r_{1}/t)\cdot (\theta_{1}\right)\right)}^{3}-6.228344\times 10^{-3}\cdot {\left(\left(r_{1}/t)\cdot (\theta_{1}\right)\right)}^{2}\\ & -3.708776\cdot ((r_{1}/t)\cdot (\theta_{1}))+5.093895\times 10^{-4} \end{array}\\ f(T/t)=-1.339099\times 10^{-1}\cdot {\left(T/t\right)}^{3}-2.209838\times 10^{-1}\cdot {\left(T/t\right)}^{2}+2.672635\cdot (T/t)+7.091184\\ f(L_{1}/t)=-3.010451\times 10^{-1}\cdot {\left(L_{1}/t\right)}^{4}+8.925728\times 10^{-1}\cdot {\left(L_{1}/t\right)}^{3}+7.371901\times 10^{-1}\cdot {\left(L_{1}/t\right)}^{2}-4.670849\cdot (L_{1}/t)+1.026529\times 10^{1}\\ \begin{array}{ll} f(L_{2}/t)= & 2.279216\cdot {\left(L_{2}/t\right)}^{0.5125725}-1.251885\times 10^{-1}\cdot {\left(L_{2}/t\right)}^{4}+4.582496\times 10^{-1}\cdot {\left(L_{2}/t\right)}^{3}-8.695745\times 10^{-1}\cdot {\left(L_{2}/t\right)}^{2}\\ & +5.580258\times 10^{-1}\cdot (L_{2}/t)+1.428608 \end{array}\\ f(H/t)=-5.287217\times 10^{1}\cdot {\left(H/t\right)}^{4}+3.348028\times 10^{1}\cdot {\left(H/t\right)}^{3}-8.163269\cdot {\left(H/t\right)}^{2}+1.102800\cdot (H/t)+6.451627\times 10^{-1}\\ f(W/t)=1.301147\times 10^{-2}\cdot {\left(W/t\right)}^{4}-9.567806\cdot {\left(W/t\right)}^{3}+2.394994\times 10^{3}\cdot {\left(W/t\right)}^{2}-2.200108\times 10^{5}\cdot (W/t)-1.3719900\times 10^{7}\\ \begin{array}{ll} f\left(\left(T/t)\cdot (L_{1}/t\right)\right)= & -3.842040\cdot {\left(\left(T/t)\cdot (L_{1}/t\right)\right)}^{0.4453254}+1.650730\times 10^{-2}\cdot {\left(\left(T/t)\cdot (L_{1}/t\right)\right)}^{3}-1.018550\times 10^{-1}\cdot {\left(\left(T/t)\cdot (L_{1}/t\right)\right)}^{2}\\ & +7.863548\times 10^{-1}\cdot \left(\left(T/t)\cdot (L_{1}/t\right)\right)+1.243387\times 10^{1} \end{array} \end{array}$$

(4) Gusset welded joints with double attachments under bending stress:

(A4) $$K_{t}=1-1.639016\times 10^{-7}\cdot f(r_{1}/t)\cdot f(\theta_{1})\cdot f\left(\left(r_{1}/t)\cdot (\theta_{1}\right)\right)\cdot f(T/t)\cdot f(L_{1}/t)\cdot f(L_{2}/t)\cdot f(H/t)\cdot f(W/t)\cdot f\left(\left(T/t)\cdot (L_{1}/t\right)\right)$$

where:

$$\begin{array}{l} f(r_{1}/t)=1.740637\cdot {\left(r_{1}/t\right)}^{-0.09991442}+2.043759\times 10^{1}\cdot {\left(r_{1}/t\right)}^{3}-1.663699\times 10^{1}\cdot {\left(r_{1}/t\right)}^{2}+6.684307\cdot (r_{1}/t)+2.890842\times 10^{-1}\\ f(\theta_{1})=3.632079\times 10^{-1}\cdot {\left(\theta_{1}\right)}^{3}-1.599956\cdot {\left(\theta_{1}\right)}^{2}+3.408799\cdot (\theta_{1})+7.312355\times 10^{-1}\\ \begin{array}{ll} f\left(\left(r_{1}/t)\cdot (\theta_{1}\right)\right)= & 6.949047\cdot {\left(\left(r_{1}/t)\cdot (\theta_{1}\right)\right)}^{1.00101058}+1.031407\times 10^{-2}\cdot {\left(\left(r_{1}/t)\cdot (\theta_{1}\right)\right)}^{3}-1.990026\times 10^{-2}\cdot {\left(\left(r_{1}/t)\cdot (\theta_{1}\right)\right)}^{2}\\ & -\;6.939134\cdot ((r_{1}/t)\cdot (\theta_{1}))+1.507149\times 10^{-3} \end{array}\\ f(T/t)=8.470214\times 10^{-2}\cdot {\left(T/t\right)}^{3}-4.864101\times 10^{-1}\cdot {\left(T/t\right)}^{2}+1.064472\cdot (T/t)+2.054082\times 10^{-1}\\ f(L_{1}/t)=-1.161963\times 10^{-1}\cdot {\left(L_{1}/t\right)}^{4}-1.881984\times 10^{-1}\cdot {\left(L_{1}/t\right)}^{3}+8.294000\times 10^{-1}\cdot {\left(L_{1}/t\right)}^{2}+2.137690\cdot (L_{1}/t)+1.034146\times 10^{1}\\ \begin{array}{ll} f(L_{2}/t)= & 8.900659\times 10^{-1}\cdot {\left(L_{2}/t\right)}^{0.06117333}+2.432168\times 10^{-1}\cdot {\left(L_{2}/t\right)}^{4}-1.108985\cdot {\left(L_{2}/t\right)}^{3}+2.38418\times 10^{-1}\cdot {\left(L_{2}/t\right)}^{2}\\ & +2.267569\cdot (L_{2}/t)+1.960294 \end{array}\\ f(H/t)=-1.015390\times 10^{1}\cdot {\left(H/t\right)}^{4}+8.206560\cdot {\left(H/t\right)}^{3}-2.220777\cdot {\left(H/t\right)}^{2}+2.913172\times 10^{-1}\cdot (H/t)+3.580056\times 10^{-1}\\ \begin{array}{ll} f(W/t)= & -8.704289\times 10^{-2}\cdot {\left(W/t\right)}^{4}+7.902012\times 10^{1}\cdot {\left(W/t\right)}^{3}-2.753614\times 10^{4}\cdot {\left(W/t\right)}^{2}+4.562486\times 10^{6}\cdot (W/t)\\ & +9.4279990\times 10^{7} \end{array}\\ \begin{array}{ll} f\left(\left(T/t)\cdot (L_{1}/t\right)\right)= & -4.027887\cdot {\left(\left(T/t)\cdot (L_{1}/t\right)\right)}^{-0.168704}-6.121183\times 10^{-3}\cdot {\left(\left(T/t)\cdot (L_{1}/t\right)\right)}^{3}+7.573732\times 10^{-2}\cdot {\left(\left(T/t)\cdot (L_{1}/t\right)\right)}^{2}\\ & -4.068440\times 10^{-1}\cdot \left(\left(T/t)\cdot (L_{1}/t\right)\right)+3.476241 \end{array} \end{array}$$

The previously given text is shown below:

## Appendix A

Formulae for calculating SCFs for gusset welded joints with single attachment and double attachments under tensile and bending stress.

(1) Gusset welded joints with single attachment under tensile stress:

(A1) $$K_{t}=1+2.75\times 10^{-5}\cdot f(r_{1}/t)\cdot f(\theta_{1}/t)\cdot f\left(\left(r_{1}/t)\cdot (\theta_{1}/t\right)\right)\cdot f(T/t)\cdot f(L_{1}/t)\cdot f(L_{2}/t)\cdot f(W/t)$$

where:

$$\begin{array}{l} f(r_{1}/t)=-1.384\cdot {\left(r_{1}/t\right)}^{0.398}+8.599\cdot {\left(r_{1}/t\right)}^{3}-7.659\cdot {\left(r_{1}/t\right)}^{2}+3.384\cdot (r_{1}/t)+0.550\\ f(\theta_{1})=-0.273\cdot {\left(\theta_{1}\right)}^{3}-0.131\cdot {\left(\theta_{1}\right)}^{2}+3.523\cdot (\theta_{1})+2.808\\ f\left(\left(r_{1}/t)\cdot (\theta_{1}\right)\right)=3.609\cdot {\left(\left(r_{1}/t)\cdot (\theta_{1}\right)\right)}^{1.001}+0.003\cdot {\left((r_{1}/t)\cdot (\theta_{1})\right)}^{3}-0.006\cdot {\left((r_{1}/t)\cdot (\theta_{1})\right)}^{2}-\;3.606\cdot ((r_{1}/t)\cdot (\theta_{1}))+4.750\times 10^{-4}\\ f(T/t)=-0.067\cdot {\left(T/t\right)}^{3}+0.281\cdot {\left(T/t\right)}^{2}-1.092\cdot (T/t)+8.586\\ f(L_{1}/t)=-0.230\cdot {\left(L_{1}/t\right)}^{4}+0.689\cdot {\left(L_{1}/t\right)}^{3}+0.158\cdot {\left(L_{1}/t\right)}^{2}-2.586\cdot (L_{1}/t)+9.521\\ f(L_{2}/t)=1.880\cdot {\left(L_{2}/t\right)}^{0.815}+1.393\cdot {\left(L_{2}/t\right)}^{4}-1.793\cdot {\left(L_{2}/t\right)}^{3}-0.983\cdot {\left(L_{2}/t\right)}^{2}+0.719\cdot (L_{2}/t)+1.448\\ f(W/t)=-0.128\cdot {\left(W/t\right)}^{4}+30.579\cdot {\left(W/t\right)}^{3}-2.505\times 10^{3}\cdot {\left(W/t\right)}^{2}+7.783\times 10^{4}\cdot (W/t)+9.862\times 10^{5} \end{array}$$

(2) Gusset welded joints with single attachment under bending stress:

(A2) $$K_{t}=1+1.090\times 10^{-4}\cdot f(r_{1}/t)\cdot f(\theta_{1}/t)\cdot f\left(\left(r_{1}/t)\cdot (\theta_{1}/t\right)\right)\cdot f(T/t)\cdot f(L_{1}/t)\cdot f(L_{2}/t)\cdot f(H/t)\cdot f(W/t)\cdot f\left(\left(T/t)\cdot (L_{1}/t\right)\right)$$

where:

$$\begin{array}{l} f(r_{1}/t)=0.222\cdot {\left(r_{1}/t\right)}^{-0.959}-9.037\cdot {\left(r_{1}/t\right)}^{3}+9.251\cdot {\left(r_{1}/t\right)}^{2}-3.923\cdot (r_{1}/t)+1.292\\ f(\theta_{1})=-0.125\cdot {\left(\theta_{1}\right)}^{3}+0.616\cdot {\left(\theta_{1}\right)}^{2}-1.372\cdot (\theta_{1})+2.713\\ f\left(\left(r_{1}/t)\cdot (\theta_{1}\right)\right)=3.673\cdot {\left(\left(r_{1}/t)\cdot (\theta_{1}\right)\right)}^{0.818}-1.709\cdot {\left((r_{1}/t)\cdot (\theta_{1})\right)}^{3}+2.460\cdot {\left((r_{1}/t)\cdot (\theta_{1})\right)}^{2}-4.563\cdot ((r_{1}/t)\cdot (\theta_{1}))-3.312\times 10^{-3}\\ f(T/t)=0.154\cdot {\left(T/t\right)}^{3}-0.223\cdot {\left(T/t\right)}^{2}-3.221\cdot (T/t)+15.852\\ f(L_{1}/t)=0.0915\cdot {\left(L_{1}/t\right)}^{4}-0.538\cdot {\left(L_{1}/t\right)}^{3}+1.217\cdot {\left(L_{1}/t\right)}^{2}-1.372\cdot (L_{1}/t)+0.955\\ f(L_{2}/t)=2.323\cdot {\left(L_{2}/t\right)}^{0.0720}+0.113\cdot {\left(L_{2}/t\right)}^{4}-0.461\cdot {\left(L_{2}/t\right)}^{3}+0.791\cdot {\left(L_{2}/t\right)}^{2}-0.817\cdot (L_{2}/t)-1.947\\ f(H/t)=-81.096\cdot {\left(H/t\right)}^{4}+48.664\cdot {\left(H/t\right)}^{3}-9.668\cdot {\left(H/t\right)}^{2}+0.906\cdot (H/t)+0.669\\ f(W/t)=-0.135\cdot {\left(W/t\right)}^{4}+37.282\cdot {\left(W/t\right)}^{3}-3.821\times 10^{3}\cdot {\left(W/t\right)}^{2}+1.793\times 10^{5}\cdot (W/t)+1.212\times 10^{6}\\ \begin{array}{ll} f\left(\left(T/t)\cdot (L_{1}/t\right)\right)= & 5.285\cdot {\left(\left(T/t)\cdot (L_{1}/t\right)\right)}^{0.754}-0.0665\cdot {\left(\left(T/t)\cdot (L_{1}/t\right)\right)}^{3}+0.580\cdot {\left(\left(T/t)\cdot (L_{1}/t\right)\right)}^{2}-\;4.395\cdot \left(\left(T/t)\cdot (L_{1}/t\right)\right)+\\ & 1.429 \end{array} \end{array}$$

(3) Gusset welded joints with double attachments under tensile stress:

(A3) $$K_{t}=1-3.220\times 10^{-8}\cdot f(r_{1}/t)\cdot f(\theta_{1}/t)\cdot f\left(\left(r_{1}/t)\cdot (\theta_{1}/t\right)\right)\cdot f(T/t)\cdot f(L_{1}/t)\cdot f(L_{2}/t)\cdot f(H/t)\cdot f(W/t)\cdot f\left(\left(T/t)\cdot (L_{1}/t\right)\right)$$

where:

$$\begin{array}{l} f(r_{1}/t)=1.683\cdot {\left(r_{1}/t\right)}^{-0.184}-17.438\cdot {\left(r_{1}/t\right)}^{3}+4.539\cdot {\left(r_{1}/t\right)}^{2}+2.538\cdot (r_{1}/t)+0.225\\ f(\theta_{1})=-0.113\cdot {\left(\theta_{1}\right)}^{3}-0.714\cdot {\left(\theta_{1}\right)}^{2}+3.800\cdot (\theta_{1})+1.308\\ \begin{array}{ll} f\left(\left(r_{1}/t)\cdot (\theta_{1}\right)\right)= & 3.712\cdot {\left(\left(r_{1}/t)\cdot (\theta_{1}\right)\right)}^{1.001}+3.489\times 10^{-3}\cdot {\left(\left(r_{1}/t)\cdot (\theta_{1}\right)\right)}^{3}+6.228\times 10^{-3}\cdot {\left(\left(r_{1}/t)\cdot (\theta_{1}\right)\right)}^{2}-3.709\cdot ((r_{1}/t)\cdot (\theta_{1}))+\\ & 5.094\times 10^{-4} \end{array}\\ f(T/t)=-0.134\cdot {\left(T/t\right)}^{3}-0.221\cdot {\left(T/t\right)}^{2}+2.673\cdot (T/t)+7.091\\ f(L_{1}/t)=-0.301\cdot {\left(L_{1}/t\right)}^{4}+0.893\cdot {\left(L_{1}/t\right)}^{3}+0.737\cdot {\left(L_{1}/t\right)}^{2}-4.671\cdot (L_{1}/t)+10.265\\ f(L_{2}/t)=2.279\cdot {\left(L_{2}/t\right)}^{0.513}-0.125\cdot {\left(L_{2}/t\right)}^{4}+0.458\cdot {\left(L_{2}/t\right)}^{3}-0.870\cdot {\left(L_{2}/t\right)}^{2}+0.558\cdot (L_{2}/t)+1.429\\ f(H/t)=-52.872\cdot {\left(H/t\right)}^{4}+33.480\cdot {\left(H/t\right)}^{3}-8.163\cdot {\left(H/t\right)}^{2}+1.103\cdot (H/t)+0.645\\ f(W/t)=0.013\cdot {\left(W/t\right)}^{4}-9.568\cdot {\left(W/t\right)}^{3}+2.395\times 10^{3}\cdot {\left(W/t\right)}^{2}-2.200\times 10^{5}\cdot (W/t)-1.372\times 10^{7}\\ \begin{array}{ll} f\left(\left(T/t)\cdot (L_{1}/t\right)\right)= & 3.842\cdot {\left(\left(T/t)\cdot (L_{1}/t\right)\right)}^{0.445}+0.0165\cdot {\left(\left(T/t)\cdot (L_{1}/t\right)\right)}^{3}-0.102\cdot {\left(\left(T/t)\cdot (L_{1}/t\right)\right)}^{2}+0.786\cdot \left(\left(T/t)\cdot (L_{1}/t\right)\right)+\\ & 12.434 \end{array} \end{array}$$

(4) Gusset welded joints with double attachments under bending stress:

(A4) $$K_{t}=1-1.639\times 10^{-7}\cdot f(r_{1}/t)\cdot f(\theta_{1}/t)\cdot f\left(\left(r_{1}/t)\cdot (\theta_{1}/t\right)\right)\cdot f(T/t)\cdot f(L_{1}/t)\cdot f(L_{2}/t)\cdot f(H/t)\cdot f(W/t)\cdot f\left(\left(T/t)\cdot (L_{1}/t\right)\right)$$

where:

$$\begin{array}{l} f(r_{1}/t)=1.741\cdot {\left(r_{1}/t\right)}^{-0.100}+20.438\cdot {\left(r_{1}/t\right)}^{3}-16.637\cdot {\left(r_{1}/t\right)}^{2}+6.684\cdot (r_{1}/t)+0.289\\ f(\theta_{1})=0.363\cdot {\left(\theta_{1}\right)}^{3}-1.600\cdot {\left(\theta_{1}\right)}^{2}+3.409\cdot (\theta_{1})+0.731\\ \begin{array}{ll} f\left(\left(r_{1}/t)\cdot (\theta_{1}\right)\right)= & 6.949\cdot {\left(\left(r_{1}/t)\cdot (\theta_{1}\right)\right)}^{1.001}+1.031\times 10^{-2}\cdot {\left(\left(r_{1}/t)\cdot (\theta_{1}\right)\right)}^{3}-1.990\times 10^{-2}\cdot {\left(\left(r_{1}/t)\cdot (\theta_{1}\right)\right)}^{2}-\;6.939\cdot ((r_{1}/t)\cdot (\theta_{1}))+\\ & 1.507\times 10^{-3} \end{array}\\ f(T/t)=0.0847\cdot {\left(T/t\right)}^{3}-0.486\cdot {\left(T/t\right)}^{2}+1.064\cdot (T/t)+0.205\\ f(L_{1}/t)=-0.116\cdot {\left(L_{1}/t\right)}^{4}-0.188\cdot {\left(L_{1}/t\right)}^{3}+0.829\cdot {\left(L_{1}/t\right)}^{2}+2.138\cdot (L_{1}/t)+10.341\\ f(L_{2}/t)=0.890\cdot {\left(L_{2}/t\right)}^{0.0612}+0.243\cdot {\left(L_{2}/t\right)}^{4}-1.109\cdot {\left(L_{2}/t\right)}^{3}+0.238\cdot {\left(L_{2}/t\right)}^{2}+2.268\cdot (L_{2}/t)+1.960\\ f(H/t)=-10.154\cdot {\left(H/t\right)}^{4}+8.207\cdot {\left(H/t\right)}^{3}-2.221\cdot {\left(H/t\right)}^{2}+0.291\cdot (H/t)+0.358\\ f(W/t)=-0.0870\cdot {\left(W/t\right)}^{4}+79.020\cdot {\left(W/t\right)}^{3}-2.754\times 10^{4}\cdot {\left(W/t\right)}^{2}+4.562\times 10^{6}\cdot (W/t)+9.428\times 10^{7}\\ \begin{array}{ll} f\left(\left(T/t)\cdot (L_{1}/t\right)\right)= & -4.028\cdot {\left(\left(T/t)\cdot (L_{1}/t\right)\right)}^{-0.169}-6.121\times 10^{-3}\cdot {\left(\left(T/t)\cdot (L_{1}/t\right)\right)}^{3}+7.574\times 10^{-2}\cdot {\left(\left(T/t)\cdot (L_{1}/t\right)\right)}^{2}-\\ & 0.407\cdot \left(\left(T/t)\cdot (L_{1}/t\right)\right)+3.476 \end{array} \end{array}$$

The authors would like to apologize for any inconvenience caused to the readers by these changes.
